# Outcomes Among Medicare Beneficiaries After Cancer Surgery in Hospitals That Subsequently Closed

**DOI:** 10.1001/jamanetworkopen.2025.53704

**Published:** 2026-01-13

**Authors:** Min-Young Kim, Douglas O. Staiger, Gabriel A. Brooks, Qianfei Wang, Sandra L. Wong, Anna N. A. Tosteson

**Affiliations:** 1 The Dartmouth Institute for Health Policy and Clinical Practice, Geisel School of Medicine at Dartmouth, Lebanon, New Hampshire; 2 Department of Economics, Dartmouth College, Hanover, New Hampshire; 3 National Bureau of Economic Research, Cambridge, Massachusetts; 4 Department of Medicine, Dartmouth Hitchcock Medical Center, Lebanon, New Hampshire; 5 Dartmouth Cancer Center, Dartmouth Hitchcock Medical Center, Lebanon, New Hampshire; 6 Department of Surgery, Dartmouth Hitchcock Medical Center, Lebanon, New Hampshire; 7 Emory School of Medicine, Atlanta, Georgia

## Abstract

**Importance:**

Hospital closures pose persistent concerns about health care access, yet the extent to which closures are associated with cancer surgical care and patient outcomes remains unknown.

**Objective:**

To examine the association between undergoing colon or lung cancer surgery in hospitals that subsequently closed and postoperative and travel outcomes among Medicare beneficiaries.

**Design, Setting, and Participants:**

This retrospective cohort study used Medicare administrative data from 2008 to 2019. A national sample of hospital closures was identified using the Provider of Service files from the Centers for Medicare & Medicaid Services. Closed cancer surgical hospitals were those performing at least 1 colon or lung cancer surgery in the period from 2008 to 2019 and that also stopped inpatient care in 2008 to 2019. Participants were Medicare fee-for-service beneficiaries who underwent colon or lung cancer surgery from 2008 to 2019. Analyses were conducted separately by cancer type. Data were analyzed from December 2023 through February 2025.

**Exposure:**

Undergoing cancer surgery at hospitals that subsequently closed.

**Main Outcomes and Measures:**

The primary outcomes were postoperative outcomes, including 90-day mortality, 90-day complications, and length of stay. Secondary outcomes were travel measures, including distance to surgical hospital and distance to the nearest alternative surgical hospital. Logistic regression was used to analyze 90-day postoperative mortality and complications, and linear regression was used to analyze length of stay. Travel measures were analyzed descriptively.

**Results:**

The total sample was 558 708 participants, with 360 564 beneficiaries (64.5%) who underwent colon cancer surgery (median [IQR] age, 77 [71-83] years; 195 862 [54.3%] female) and 198 144 beneficiaries (35.5%) who underwent lung cancer surgery (median [IQR] age, 73 [69-78] years; 102 418 [51.7%] female) from 2008 to 2019. Of those, 6018 beneficiaries (1.7%) who underwent colon cancer surgery and 1938 beneficiaries (1.0%) who underwent lung cancer surgery underwent those surgical procedures at hospitals that subsequently closed. Beneficiaries treated at hospitals that subsequently closed were more often dually eligible (colon: 1047 [17.4%] closing vs 37228 [10.5%] nonclosing; lung: 234 [12.1%] closing vs 14426 [7.4%] nonclosing) and Black, Hispanic, or other race (ie, American Indian or Alaska Native, Asian, other, and unknown) (colon: 1450 [24.1%] closing vs 53640 [15.1%] nonclosing; lung: 388 [20.0%] closing vs 22048 [11.2%] nonclosing), with urgent admission (colon: 2559 [42.5%] closing vs 123830 [34.9%] nonclosing; lung: 228 [11.8%] closing vs 13394 [6.8%] nonclosing) than those treated at hospitals that did not close. Most beneficiaries bypassed their nearest hospital, but the majority treated at their nearest hospital that subsequently closed (colon, 1967 beneficiaries [79.0%]; lung, 465 beneficiaries [90.6%]) had an alternative surgical hospital within a 15-minute driving distance. Undergoing surgery at hospitals that subsequently closed was significantly associated with higher likelihood of 90-day mortality for colon cancer (adjusted odds ratio [aOR] 1.11; 95% CI, 1.01-1.22) and 90-day complications for both cancer types (colon aOR, 1.10; 95% CI, 1.01-1.21; lung aOR, 1.43, 95% CI, 1.17-1.76). The odds ratio for 90-day mortality after lung cancer surgery was not statistically significant, 1.26 (95% CI, 0.96-1.64). Lengths of stay were similar for both cancers.

**Conclusions and Relevance:**

In this cohort study, undergoing colon and lung cancer surgery at hospitals that subsequently closed was associated with worse postoperative outcomes, but most beneficiaries treated at their nearest hospital had a nearby alternative hospital, suggesting that hospital closures may improve postoperative outcomes for cancer surgery, with minimal increase in travel burden, by directing patients to nearby, better-performing hospitals.

## Key Points

**Question:**

What were the postoperative and travel outcomes among Medicare beneficiaries who underwent colon or lung cancer surgery at hospitals that subsequently closed?

**Findings:**

In this cohort study of 558 708 Medicare beneficiares, undergoing cancer surgery at hospitals that subsequently closed was significantly associated with worse postoperative mortality and complications than undergoing surgery at hospitals that did not close. Most beneficiaries treated at their nearest hospital that subsequently closed had another surgical hospital nearby.

**Meaning:**

These findings suggest that hospital closures may be associated with improved cancer surgical outcomes, with minimal increase in beneficiaries’ travel burden.

## Teaser

This cohort study investigates postoperative and travel outcomes among Medicare beneficiaries with colon or lung cancer who underwent surgery in hospitals that subsequently closed.

## Introduction

Since 2010, more than 300 hospitals have closed across the US, and an additional 700 are at risk of closure in the next decade.^1,2^ Various factors lead to hospital closures, including financial instability from declining patient volumes and inadequate reimbursements, for-profit ownership, and the general industry shift toward low-cost, high-value care.^3,4,5,6,7,8^

Recently, rural hospital closures have garnered considerable attention because they can be detrimental to local health care availability and the economic well-being of their communities.^9,10,11,12,13,14^ The predominant focus on rural hospital closures, however, raises questions regarding how hospital closures affect specialized surgical care, such as cancer surgery. Surgical resection is an essential treatment for many types of cancer.^15,16^ Generally, cancer surgery is concentrated at nonrural hospitals that are less likely to close than rural hospitals.^17,18^ Most patients bypass their nearest hospital to undergo surgery at higher-quality hospitals, with the degree of bypassing increasing with rurality.^19,20,21^ Understanding how hospital closures are associated with cancer surgical care has important policy implications for access to and outcomes of specialized surgical services.

In this study, we investigate the extent to which hospital closures were associated with cancer surgical care among Medicare beneficiaries from 2008 to 2019. To establish the hospital landscape, we first describe cancer surgical hospitals that closed from 2008 to 2019. Then, we explore 2 questions. First, what were the characteristics of beneficiaries who underwent cancer surgery at hospitals that subsequently closed (hereafter, closing hospitals)? Second, what was the association between undergoing cancer surgery at closing hospitals and beneficiaries’ travel and postoperative outcomes? Our study examined colon and lung cancer because they are 2 common types of cancer treated by surgery and thus have broader implications for cancer surgical care.^15,22^ Moreover, hospital closures may affect colon and lung cancer differently. Colon cancer surgery is more widely available at smaller hospitals because it can be performed emergently and by a general surgeon. Lung cancer surgery is concentrated at fewer, larger hospitals because it is rarely emergent and requires specially trained surgeons.

## Methods

This cohort study was reviewed and approved by the Dartmouth College Committee for the Protection of Human Subjects and informed consent was waived because the research was determined to pose minimal risk to participants. It followed the Strengthening the Reporting of Observational Studies in Epidemiology (STROBE) reporting guidelines for cohort studies.^23^

### Study Population and Data

We used 100% Medicare inpatient and Part B claims data to identify fee-for-service (FFS) beneficiaries who received a diagnosis of nonmetastatic colon or lung cancer and had an inpatient stay for cancer resection surgery (eg, partial colectomy or pulmonary lobectomy) from 2008 to 2019 (diagnosis and procedure codes are shown in eTable 1 in Supplement 1). We restricted to beneficiaries who resided in the US, were aged 65 years or older at the time of surgery, and had FFS Parts A and B coverage during 3 months following surgery or up to date of death. We retained the first-occurring cancer resection surgery for each beneficiary (sample flowchart is shown in the eFigure in Supplement 1).

### Identifying Hospital Closures

Cancer surgical hospitals included short-stay acute-care hospitals or critical access hospitals (CAHs) that performed at least 1 cancer surgery in the 2008 to 2019 period. Of these, we defined closures as those that stopped providing Medicare inpatient care in 2008 to 2019. Using the 2006 to 2020 Provider of Service files from the Centers for Medicare & Medicaid Services,^24^ we first identified all unique hospitals using the Centers for Medicare & Medicaid Services certification numbers (CCNs). We then restricted to short-stay, acute-care hospital or CAH CCNs, reconciled data inconsistencies across years, and grouped CCNs that converted, consolidated, or merged into a single facility. We classified facilities as closed if they converted into noninpatient facilities such as skilled nursing facilities or long-term care hospitals, or if their CCN had a termination date without further conversion or consolidation. We manually verified 300 CCNs (5%) using news reports, hospital websites, and government documents, as done in prior studies.^9,10^ For hospitals identified as closed, we checked that their Medicare admissions fell to zero within 2 years after the closure year. If admissions continued, we reassigned the closure year to the year of zero admission^25^ (closure identification details are shown in the eMethods in Supplement 1). The final hospital samples, created separately for colon and lung cancer, comprised hospitals that performed at least 1 FFS cancer surgery from 2008 to 2019.

### Measures and Outcomes

#### Beneficiary Characteristics

From the denominator files, we extracted age at surgery, sex, race and ethnicity (categorized as Black, Hispanic, White, and other [ie, American Indian or Alaska Native, Asian, other, and unknown]), full-dual eligibility during 12 months prior to surgery, disability status from the original Medicare entitlement reason, date of death, rurality of residence based on Rural-Urban Commuting Area codes (1-3, metropolitan; 4-6, micropolitan; 7-10, small town or rural),^26^ and hospital service area (HSA) based on zip codes. Data on race and ethnicity are included because beneficiaries’ likelihood of undergoing surgical procedures at closing hospitals may differ across racial and ethnic categories, which can have important implications for surgical outcomes. We used claims for urgency of admission and Elixhauser comorbidities during 12 months prior to surgery.^27,28,29,30^ We used the 2015 Area Deprivation Index national rankings (range 1-100)^31,32^ averaged at the zip code level and categorized those with an average of 80 or more as areas experiencing high levels of socioeconomic deprivation.

#### Hospital Characteristics

We used the 2008 to 2019 American Hospital Association Annual Hospital Surveys to obtain hospital bed count, teaching status, whether the hospital provided oncology services, and whether the hospital had a high-quality cancer surgery program recognized by the American College of Surgeons.^33^ Critical access hospitals were identified using American Hospital Association reporting or the CCN facility type. We assigned rurality on the basis of Rural-Urban Commuting Area codes (1-3, metropolitan; other, nonmetropolitan) and determined hospital census region and HSA. We counted surgical beneficiaries for annual surgical volume. Yearly data were aggregated to the hospital level by assigning the mode for categorical or mean for continuous variables.

#### Travel Outcomes

We calculated driving distance and time between beneficiary zip code centroid to all surgical hospitals using ArcGIS Pro software version 3.3.1 (Esri) and ranked hospitals according to their proximity to the zip code centroid. One-way travel times were capped at 6 hours. We identified beneficiaries who remained in their HSA for surgery and those treated at their nearest hospital. For beneficiaries treated at their nearest hospital, we calculated additional travel time to the next nearest surgical hospital.

#### Postoperative Outcomes

We examined 90-day mortality, 90-day nondeath complications, and length of stay, all obtained from claims. The list of complications is shown in eTable 2 in Supplement 1.

### Statistical Analysis

To establish the landscape of cancer surgical hospital closures, we examined closure trend by graphing the number of closures overall and by cancer surgical type and mapped the geographic distribution of hospital closures. We then compared the hospital characteristics by closure status using χ^2^ tests for categorical variables and Wilcoxon rank-sum tests for skewed continuous variables.

#### Primary Analysis

For the main analysis, we described beneficiary characteristics and travel measures by hospital closure status using the same tests as hospital characteristics. For the association between undergoing surgery at closing hospitals and postoperative outcomes, we used logistic regression for 90-day postoperative mortality and complications and linear regression for length of stay. For each outcome, we controlled for surgery year and all beneficiary characteristics with SEs clustered at the hospital level. Statistical significance was determined at 2-sided *P* < .05. We used R Studio version 4.3.3 (R Project for Statistical Computing) for mapping and Stata/MP version 18.0 (StataCorp) for statistical analyses.

#### Sensitivity Analysis

Our exposed group included beneficiaries who underwent surgery at a closing hospital over 12 years. Although this maximized the sample size of the exposed group, it called into question the temporal relevance of surgical procedures that occurred many years before closure. To address this, we performed a sensitivity analysis by restricting the exposed group to beneficiaries who underwent surgery within 2, 4, and 6 years prior to closure and repeating the regression analyses. For each regression, we assessed power by estimating the minimum detectable effect size using the observed exposed and unexposed sample sizes, an α of .05, and a power of 0.80.

## Results

The total sample size of participants was 558 708 Medicare beneficiaries, with 360 564 (64.5%) who underwent colon cancer surgery (median [IQR] age, 77 [71-83] years; 195 826 [54.3%] female) and 198 144 (35.5%) who underwent lung cancer surgery (median [IQR] age 73, [69-78] years; 102 418 [51.7%] female) from 2008 to 2019. Additional beneficiary characteristics are shown in Table 1. During the study period, there were a total of 3965 hospitals performing colon cancer surgical procedures and 2182 hospitals performing lung cancer surgical procedures. Characteristics of surgical hospitals are shown in Table 2.

**Table 1.** Characteristics of Cancer Surgical Beneficiaries by Hospital Closure Status and Cancer Type, 2008 to 2019

Characteristic	Beneficiaries with colon cancer, No. (%)			Beneficiaries with lung cancer, No. (%)		
	Nonclosing hospital (n = 354 546)	Closing hospital (n = 6018)	*P* value^a^	Nonclosing hospital (n = 196 206)	Closing hospital (n = 1938)	*P* value^a^
**Patient characteristics**						
Age group, y						
65-74	140 474 (39.6)	2300 (38.2)	.002	115 171 (58.7)	1079 (55.7)	.001
75-84	144 085 (40.6)	2422 (40.3)		73 417 (37.4)	757 (39.1)	
≥85	69 987 (19.7)	1296 (21.5)		7618 (3.9)	102 (5.3)	
Sex						
Female	192 504 (54.3)	3322 (55.2)	.16	101 448 (51.7)	970 (50.1)	.15
Male	162 042 (45.7)	2696 (44.8)		94 758 (48.3)	968 (49.9)	
Race						
Black	27 958 (7.9)	773 (12.8)	<.001	10 720 (5.5)	173 (8.9)	<.001
Hispanic	13 590 (3.8)	422 (7.0)		4809 (2.5)	139 (7.2)	
Other^b^	12 092 (3.4)	255 (4.2)		6519 (3.3)	76 (3.9)	
White	300 906 (84.9)	4568 (75.9)		174 158 (88.8)	1550 (80.0)	
Dually eligible^c^	37 228 (10.5)	1047 (17.4)	<.001	14 426 (7.4)	234 (12.1)	<.001
Disabled	31 559 (8.9)	610 (10.1)	<.001	22 732 (11.6)	226 (11.7)	.92
Urgent admission^d^	123 830 (34.9)	2559 (42.5)	<.001	13 394 (6.8)	228 (11.8)	<.001
Comorbidities^e^						
0	55 566 (15.7)	931 (15.5)	.02	18 504 (9.4)	127 (6.6)	<.001
1-2	130 864 (36.9)	2113 (35.1)		74 931 (38.2)	719 (37.1)	
3-4	90 813 (25.6)	1615 (26.8)		61 999 (31.6)	632 (32.6)	
≥5	77 303 (21.8)	1359 (22.6)		40 772 (20.8)	460 (23.7)	
Rurality^f^						
Metropolitan	263 149 (74.2)	4642 (77.1)	<.001	154 190 (78.6)	1614 (83.3)	<.001
Micropolitan	45 663 (12.9)	828 (13.8)		22 092 (11.3)	196 (10.1)	
Small town or rural	45 734 (12.9)	548 (9.1)		19 924 (10.2)	128 (6.6)	
Area Deprivation Index ≥80	31 622 (8.9)	684 (11.4)	<.001	13 943 (7.1)	138 (7.1)	.98
**Travel to surgical hospital**						
Surgery in Hospital Service Area	212 753 (60.0)	3974 (66.0)	<.001	86 682 (44.2)	1099 (56.7)	<.001
Travel distance, median (IQR), miles	10 (4-25)	7 (3-15)	<.001	17 (7-43)	10 (5-30)	<.001
Travel time, median (IQR), min	18 (10-34)	14 (8-24)	<.001	26 (14-53)	19 (12-37)	<.001
Surgery at nearest hospital	138 416 (39.1)	2489 (41.4)	<.001	55 343 (28.2)	513 (26.5)	.09
If surgery at nearest hospital						
Had another hospital within 15 min	97 389 (70.4)	1967 (79.0)	<.001	40 923 (73.9)	465 (90.6)	<.001
Time to next nearest hospital, median (IQR), min	8 (3-18)	6 (2-12)	<.001	7 (2-16)	4 (1-8)	<.001

^a^ We calculated *P* values using χ^2^ test for categorical variables and Wilcoxon rank-sum test for skewed continuous variables.

^b^ Other race includes American Indian or Alaska Native, Asian, other, and unknown.

^c^ Dual eligibility was determined according to 12 months prior to surgery.

^d^ Urgent admission category included both urgent and emergent admissions.

^e^ Comorbidities were determined according to the Elixhauser comorbidities algorithm.

^f^ Rurality of residence was based on the Rural-Urban Commuting Area codes (1-3, metropolitan; 4-6, micropolitan; and 7-10, small town or rural).

**Table 2.** Cancer Surgical Hospital Characteristics by Closure Status, 2008 to 2019

Characteristic	Colon cancer surgical hospitals, No. (%)			Lung cancer surgical hospitals, No. (%)		
	Nonclosing (n = 3698)	Closing (n = 267)	*P* value^a^	Nonclosing (n = 2074)	Closing (n = 108)	*P* value^a^
Bed count size						
<100	1512 (40.9)	129 (48.3)	.07	251 (12.1)	26 (24.1)	<.001
100-299	1399 (37.8)	94 (35.2)		1048 (50.5)	57 (52.8)	
≥300	787 (21.3)	44 (16.5)		775 (37.4)	25 (23.1)	
Annual surgical volume, median (IQR), No. of beneficiaries	5 (2-11)	2 (1-5)	<.001	4 (2-10)	2 (1-4)	<.001
For-profit ownership	639 (17.3)	107 (40.1)	<.001	382 (18.4)	38 (35.2)	<.001
Nonteaching hospital	273 (7.4)	30 (11.2)	<.001	266 (12.8)	15 (13.9)	.01
Provides oncology services	2450 (66.3)	94 (35.2)	<.001	1774 (85.5)	57 (52.8)	<.001
American College of Surgeons–approved cancer program	1352 (36.6)	38 (14.2)	<.001	1232 (59.4)	32 (29.6)	<.001
Critical access hospital	797 (21.6)	13 (4.9)	<.001	58 (2.8)	0 (0.0)	.08
Metropolitan	2164 (58.5)	194 (72.7)	<.001	1718 (82.8)	100 (92.6)	.008
Census region						
Northeast	516 (14.0)	49 (18.4)	<.001	369 (17.8)	26 (24.1)	.09
Midwest	1116 (30.2)	48 (18.0)		495 (23.9)	21 (19.4)	
South	1345 (36.4)	130 (48.7)		771 (37.2)	46 (42.6)	
West	721 (19.5)	40 (15.0)		439 (21.2)	15 (13.9)	

^a^ *P* values were calculated using χ^2^ test for categorical variables and Wilcoxon rank-sum test for skewed continuous variables.

### Hospital Closure Trend and Characteristics

**Figure 1.**
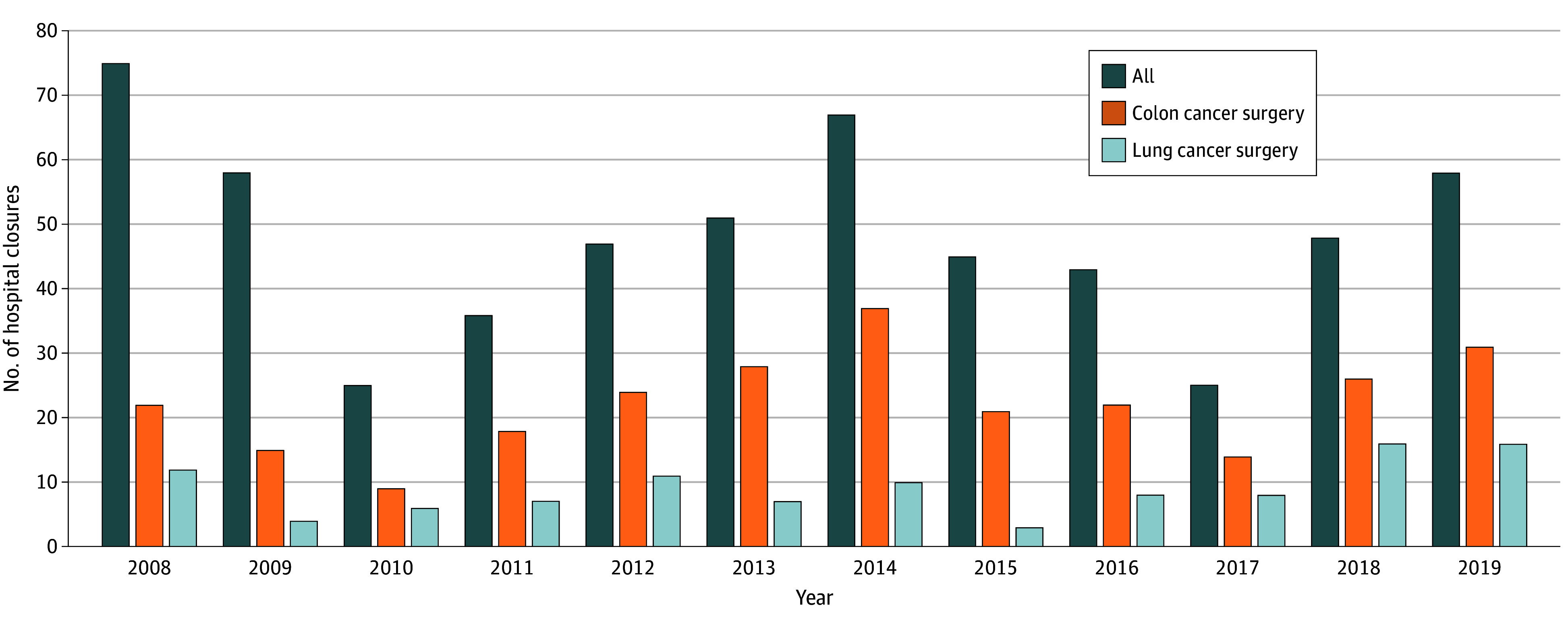
Number of Hospital Closures, Overall and by Cancer Surgery Type Graph shows authors’ analysis of the Provider of Service Files from the Centers for Medicare & Medicaid Services and Medicare fee-for-service claims data.

**Figure 2.**
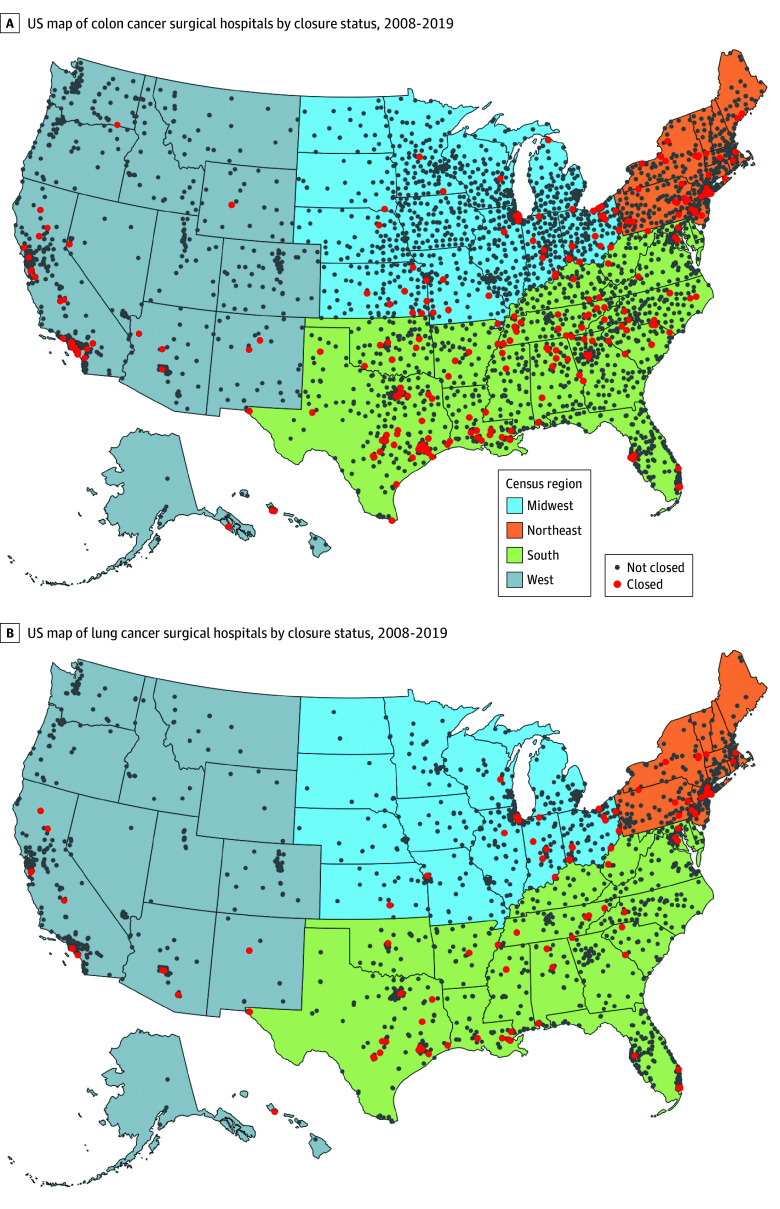
US Map of Cancer Surgical Hospitals by Closure Status, 2008 to 2019

From 2008 to 2019, among 3965 hospitals performing colon cancer surgery, 267 (6.7%) closed; among 2182 hospitals performing lung cancer surgery, 108 (4.9%) closed. A total of 578 hospitals closed, 267 of which (46.2%) performed colon cancer surgery and 108 of which (18.7%) performed lung cancer surgery (Figure 1), but closing hospitals performed only 6018 of 360 564 colon procedures (1.7%) and 1938 of 198 144 lung procedures (1.0%) during the study period. Closing cancer surgical hospitals were concentrated in the Northeast and the South around major cities, including New York, New York; Philadelphia, Pennsylvania; Cleveland, Ohio; Houston, Texas; Baton Rouge, Louisiana; and Los Angeles, California (Figure 2A and 2B).

For both cancers, closing hospitals were significantly more likely to be small, for-profit, nonteaching, and nononcology specialty hospitals in metropolitan areas than hospitals that did not close. Closing hospitals were more likely to have fewer than 100 beds with half the median annual surgical volume than hospitals that did not close (Table 2). Closing hospitals were more likely to report for-profit ownership (colon: 107 [40.1%] vs 639 [17.3%]; lung: 38 [35.2%] vs 382 [18.4%]) and less likely to be teaching hospitals. Closing hospitals were much less likely to have an American College of Surgeons–approved cancer program (colon: 38 [14.2%] vs 1352 [36.6%]; lung: 32 [29.6%] vs 1232 [59.4%]). Finally, closing hospitals were less likely to be CAHs and more likely to be located in metropolitan areas (colon: 194 [72.7%] vs 2164 [58.5%]; lung: 100 [92.6%] vs 1718 [82.8%]).

### Beneficiary Characteristics

Beneficiaries treated at closing hospitals were more likely to be older (colon: ≥85 years old, 1296 [21.5%] at closing hospitals vs 69 987 [19.7%] at nonclosing hospitals; lung: ≥85 years old, 102 [5.3%] closing vs 7618 [3.9%] nonclosing); Black, Hispanic, or other race (colon: 1450 [24.1%] closing vs 53 640 [15.1%] nonclosing; lung: 388 [20.0%] closing vs 22 048 [11.2%] nonclosing); and dually eligible (colon: 1047 [17.4%] closing vs 37 228 [10.5%] nonclosing; lung: 234 [12.1%] closing vs 14 426 [7.4%] nonclosing) than beneficiaries at nonclosing hospitals (Table 1). Beneficiaries treated at closing hospitals were significantly more likely to have an urgent admission (colon: 2559 [42.5%] closing vs 123 830 [34.9%] nonclosing; lung: 228 [11.8%] closing vs 13 394 [6.8%] nonclosing) and a higher number of comorbidities (≥5 for colon: 1359 closing [22.6%] vs 77 303 [21.8%] nonclosing; ≥5 for lung: 460 [23.7%] closing vs 40 772 [20.8%] nonclosing). Beneficiaries treated at closing hospitals tended to reside in metropolitan zip codes (colon: 4642 [77.1%] closing vs 263 149 [74.2%] nonclosing; lung: 1614 [83.3%] closing vs 154 190 [78.6%] nonclosing), with colon cancer beneficiaries residing in higher deprivation areas (684 [11.4%] closing vs 31 622 [8.9%] nonclosing).

### Travel Patterns

For both cancers, beneficiaries treated at closing hospitals were significantly more likely to undergo surgery in their HSA (colon: 3974 [66.0%] at closing hospitals vs 212 753 [60.0%] at nonclosing hospitals; lung: 1099 [56.7%] closing vs 86 682 [44.2%] nonclosing) and travel less for surgery (median [IQR] distance traveled for colon: 7 [3-15] miles for closing hospitals vs 10 [4-25] miles for nonclosing hospitals; lung: 10 [5-30] miles for closing hospitals vs 17 [7-43] miles for nonclosing hospitals) than beneficiaries treated at hospitals that did not close. Most beneficiaries bypassed their nearest hospital for surgery. However, among those treated at their nearest hospital, more than 70% had an alternative surgical hospital available within 15 minutes, and this proportion was higher for beneficiaries treated at closing hospitals (colon: 1967 [79.0%] closing vs 97 389 [70.4%] nonclosing; lung: 465 [90.6%] closing vs 40 923 [73.9%] nonclosing). For beneficiaries treated at their nearest hospital, the median additional driving time to the next nearest surgical hospital was 6 to 8 minutes for colon and 4 to 7 minutes for lung.

### Postoperative Outcomes

Undergoing surgery at closing hospitals was significantly associated with worse postoperative outcomes for both cancers. For colon cancer, surgery at closing hospitals was associated with higher odds of 90-day mortality (adjusted odds ratio [aOR], 1.11; 95% CI, 1.01-1.22) and 90-day complications (aOR, 1.10; 95% CI, 1.01-1.21) (Table 3). For lung cancer, surgery at closing hospitals was associated with higher odds of 90-day complications (aOR, 1.43; 95% CI, 1.17-1.76). The odds ratio for 90-day mortality after lung surgery was 1.26 (95% CI, 0.96-1.64), indicating a result that was not statistically significant.

**Table 3.** Association Between Undergoing Surgery at a Closing Hospital and Postoperative Outcomes, by Cancer

Type of cancer and outcomes	Summary statistics, No. (%)		Regression coefficient (95% CI)^a^	
	At nonclosing hospital	At closing hospital	Unadjusted	Adjusted^b^
Colon				
90-d Mortality	35 349 (10.0)	765 (12.7)	1.32 (1.22 to 1.42)^c^	1.11 (1.01 to 1.22)^d^
90-d Complications	164 888 (46.5)	3190 (53.0)	1.30 (1.23 to 1.37)^c^	1.10 (1.01 to 1.21)^d^
Length of stay, mean (SD), d	8.9 (7.2)	10.1 (7.6)	1.18 (1.00 to 1.37)^c^	0.11 (−0.27 to 0.50)
Lung				
90-d Mortality	11 249 (5.7)	170 (8.8)	1.58 (1.35 to 1.85)^c^	1.26 (0.96 to 1.64)
90-d Complications	81 357 (41.5)	1052 (54.3)	1.68 (1.53 to 1.83)^c^	1.43 (1.17 to 1.76)^c^
Length of stay, mean (SD), d	6.9 (6.2)	8.3 (6.6)	1.36 (1.08 to 1.64)^c^	0.65 (−0.25 to 1.56)

^a^ The regression coefficients are odds ratios for mortality and complications and change in days for length of stay.

^b^ Adjusted models controlled for beneficiary characteristics, including age, sex, race, dual eligibility, disability status, Elixhauser comorbidities, admission urgency, zip code rurality, and zip code Area Deprivation Index, and the year of surgery with SE clustered at the hospital level.

^c^ *P* < .001.

^d^ *P* < .05.

### Sensitivity Analysis

Although sensitivity analysis showed similar associations as the main results, restricting the exposed group to beneficiaries who underwent surgery within 2, 4, and 6 years prior to closure reduced the sample size and power to detect effects (sensitivity regression results are shown in eTables 3-8 in Supplement 1). For nearly all sensitivity regressions, the minimum detectable effect size was above the estimated effect size, indicating insufficient power. One exception was 90-day complications for lung cancer surgery, which showed statistically significant aORs of similar magnitude as the main analysis (regression table is shown in eTable 7 in Supplement 1).

## Discussion

In this cohort study, we briefly described the characteristics of closing cancer surgical hospitals and examined postoperative and travel outcomes of Medicare FFS beneficiaries who underwent colon or lung cancer surgery at hospitals that subsequently closed in the period from 2008 to 2019. Closing hospitals were typically small, for-profit, nonteaching, and nononcology specialty hospitals, largely located in metropolitan areas. Although few beneficiaries were treated at closing hospitals, they were more often older, dually eligible, and Black, Hispanic, or other race, with a higher number of comorbid conditions and urgent admissions compared with beneficiaries treated at hospitals that did not close. Undergoing surgery at closing hospitals was associated with higher odds of experiencing adverse postoperative outcomes, but the majority of beneficiaries treated at their nearest hospital had an alternative surgical hospital nearby.

Our study offers several important takeaways. First, our study adds nuance to understanding how hospital closures affect access and outcomes for cancer surgery. At first glance, our findings appear to highlight disparities in access and outcomes for a small but vulnerable population undergoing surgery at closing hospitals, raising concerns about their potential lack of alternative surgical hospitals. Interestingly, more than 70% of beneficiaries treated at their nearest hospital had another cancer surgical hospital within 15 minutes, and this proportion was higher for beneficiaries treated at their nearest hospital that subsequently closed. This suggests limited impact of closures on overall geographic access to surgical care. Taken together, our findings suggest that some beneficiaries may benefit from closures by being reallocated to higher-performing hospitals with minimal increase in travel burden.

Second, our findings diverge from the common perception that specialty surgical hospitals face low risk of closure and that hospital closures are a rural phenomenon. Notably, we found that nearly one-half of all closing hospitals provided colon cancer surgery and one-fifth provided lung cancer surgery, indicating that cancer surgical hospitals, even those equipped to perform complex lung cancer surgery, were susceptible to closure. Moreover, closing cancer surgical hospitals were predominantly located in metropolitan areas, an expected finding as cancer surgery is concentrated at nonrural hospitals.^18,34^ Urban closures have gained much less attention from researchers and policymakers because urban patients generally have multiple hospital options within close proximity.^35^ Emerging research, however, recognizes the outsized number of urban closures, suggesting that they require further scrutiny, especially in light of recent high-profile urban hospital closures.^35,36,37,38,39,40^ Similarly, our study highlights the role that urban closures can play in changing the landscape of hospital availability for cancer surgical care.

Third, our study contributes to the literature by underscoring how differences in the urgency of medical conditions can influence the impact of hospital closures. Unlike studies examining emergent conditions like heart attack and stroke that show that hospital closures worsen access and mortality,^9,10,41^ our study shows that hospital closures may improve outcomes by reallocating patients to higher-performing hospitals. This is primarily because most cancer surgical procedures are nonurgent, and patients have more agency in choosing hospital for care, often bypassing closer, lower-quality hospitals.^19,20^ Our findings are consistent with a study by Fischer and colleagues,^42^ who found that obstetric unit closures led to slightly improved maternal and perinatal outcomes because of longer-term planned care at more distant but higher-quality hospitals. Considering the urgency of care required for a given condition can inform how the impact of hospital closures might vary across different types of cancers or surgical conditions. For example, hospital closures would have different effects on emergent colon cancer surgical procedures performed for perforations than lung cancer surgery in general that are rarely emergent.

Finally, we contribute to the ongoing policy debate around hospital closures across the US. On one hand, hospital closures, particularly of low-volume hospitals, can facilitate regionalization efforts by shifting care to higher-volume centers, which can lead to better patient outcomes.^17,43^ Our results support organizations like the Leapfrog Group, which recommend minimum surgical volumes to promote high-quality care.^44^ On the other hand, most policy responses to hospital closures—such as enhanced reimbursement for safety-net or rural hospitals and government bailouts—have largely relied on one-size-fits-all strategies based on the assumption that all hospital closures are harmful. However, this study and other studies collectively show that the impact of closures is not uniform. For cancer surgical care, hospital closures could benefit patients by redirecting care from low-performing to higher-performing hospitals, potentially improving outcomes. Implications for hospital closures also extend beyond health services into local economies.^11,14^ Our findings call for policymakers to rigorously evaluate hospital closures using a broader set of health care and economic metrics and prioritize investment in hospitals whose closure would pose the greatest detriment to both patients and their communities.

### Limitations

This study has limitations that should be mentioned. First, we used Medicare administrative data, which lack detailed clinical information like cancer stage. Without cancer stage, our results may partly reflect differences in case mix rather than differences in outcomes by closure status. Prior studies, however, found that controlling for detailed clinical factors in cancer registry data had little impact on measuring hospital performance on short-term outcomes, suggesting limited role of cancer stage in assessing surgical outcome differences between hospitals by closure status.^45,46,47,48^ Second, our analysis was restricted to Medicare FFS patients, so the findings may not generalize to younger patients or non-FFS populations. External populations may show systematically different results according to the restrictiveness of their hospital networks and likelihood of receiving timely diagnosis and treatment. Third, although we followed the methods used in a prior study,^10^ we could have misidentified some closures as nonclosed because we aimed to minimize manual updates to preserve reproducibility. This would have biased our results toward the null. Fourth, our analyses demonstrate associations and should not be interpreted as showing causality. Notably, our study design cannot disentangle whether the associations reflect valid differences in surgical quality between hospitals by closure status or preexisting unmeasured quality or patient selection issues unrelated to closure. Future research should employ quasi-experimental methods to establish temporality and investigate postclosure effects on cancer surgical access and outcomes.

## Conclusions

In this study, patients who received cancer surgery at hospitals that subsequently closed had higher risks of 90-day mortality for colon cancer surgery and of 90-day complications for colon and lung cancer surgery. Most patients lived within 15 minutes of another hospital. Hospital closures for colon and lung cancer surgery were largely an urban phenomenon, affecting few beneficiaries who were older, low income, clinically vulnerable, and Black, Hispanic, or other races. Closures may improve postoperative outcomes with minimal impact on travel burden by directing patients to nearby hospitals with better performance. Policymakers should rigorously evaluate closures using multiple metrics and prioritize investments in hospitals whose closure would pose the greatest harm to patients and their communities.

## Notes

Supplement 1. eMethods. Identifying hospital closures eTable 1. Diagnosis and procedure codes for identifying colon and lung cancer surgical Medicare FFS beneficiaries eTable 2. List of complications following cancer-directed surgery eTable 3. Colon—Sensitivity analysis for 90-day mortality eTable 4. Colon—Sensitivity analysis for 90-day complications eTable 5. Colon—Sensitivity analysis for length of stay eTable 6. Lung—Sensitivity analysis for 90-day mortality eTable 7. Lung—Sensitivity analysis for 90-day complications eTable 8. Lung—Sensitivity analysis for length of stay eFigure. Sample flowchart

Supplement 2. Data Sharing Statement
