# Supplementary material for: Outcomes Among Medicare Beneficiaries After Cancer Surgery in Hospitals That Subsequently Closed
Source: JAMA Netw Open. 2026 Jan 13;9(1):e2553704. doi: 10.1001/jamanetworkopen.2025.53704 (PMC12801090; doi:10.1001/jamanetworkopen.2025.53704)
Supplement: Supplement 1. — eMethods. Identifying hospital closures eTable 1. Diagnosis and procedure codes for identifying colon and lung cancer surgical Medicare FFS beneficiaries eTable 2. List of complications following cancer-directed surgery eTable 3. Colon—Sensitivity analysis for 90-day mortality eTable 4. Colon—Sensitivity analysis for 90-day complications eTable 5. Colon—Sensitivity analysis for length of stay eTable 6. Lung—Sensitivity analysis for 90-day mortality eTable 7. Lung—Sensitivity analysis for 90-day complications eTable 8. Lung—Sensitivity analysis for length of stay eFigure. Sample flowchart [file jamanetwopen-e2553704-s001.pdf]

Supplemental Online Content

Kim M, Staiger DO, Brooks GA, Wang Q, Wong SL, Tosteson AA. Outcomes among Medicare beneficiaries after cancer surgery in hospitals that subsequently closed. *JAMA Netw Open.* 2026;9(1):e2553704. doi:10.1001/jamanetworkopen.2025.53704

eMethods. Identifying hospital closures

eTable 1. Diagnosis and procedure codes for identifying colon and lung cancer surgical Medicare FFS beneficiaries

eTable 2. List of complications following cancer-directed surgery

eTable 3. Colon—Sensitivity analysis for 90-day mortality

eTable 4. Colon—Sensitivity analysis for 90-day complications

eTable 5. Colon—Sensitivity analysis for length of stay

eTable 6. Lung—Sensitivity analysis for 90-day mortality

eTable 7. Lung—Sensitivity analysis for 90-day complications

eTable 8. Lung—Sensitivity analysis for length of stay

eFigure. Sample flowchart

This supplemental material has been provided by the authors to give readers additional information about their work.

© 2026 Kim M et al. *JAMA Network Open*.

eMethods. Identifying hospital closures

We used the following steps to identify hospital closures for cancer surgical care.

1. Identifying the universe of hospitals

   Using the 2006-2020 Provider of Service files from the Centers for Medicare and Medicaid Services (CMS), we extracted all unique hospital IDs (CMS certification numbers [CCNs]) that were ever certified to provide care for Medicare patients. We restricted to CCNs that were (1) located in 50 states and the District of Columbia and (2) were either short-stay acute-care hospitals or critical access hospitals.

2. Aggregating CCNs to the facility level

   We aggregated CCNs to the hospital level by grouping linked CCNs. This step allowed us to account for facility conversions, consolidations, or mergers, which get assigned unique CCNs even though the facility remains unchanged.

3. Determining hospital closure status and year

   We used the CMS termination date and termination code to determine facility closure status and closure year. When termination date and codes were inconsistent, we prioritized the termination date. We manually verified the closure status for 300 facilities using newspaper reports, hospital websites, government documents, and general Google searches. We restricted to all facilities that had not closed by January 1, 2008.

4. Verifying using Medicare admissions

   We ensured that all facilities in the sample had at least 1 Medicare fee-for-service (FFS) hospital admissions in 2008-2019 using the Medicare Provider Analysis and Review files. We also checked that for all hospitals identified as closed, their Medicare admissions fell to zero within two years of the identified closure year. If admissions continued beyond two years, we reset the closure year to the year of zero admission.

5. Finalizing the hospital sample

   We merged the hospitals identified above to the cancer surgical beneficiary samples, separately by cancer. The matched hospitals represented the final cancer surgical hospitals included in the analysis.

© 2026 Kim M et al. *JAMA Network Open*.

eTable 1. Diagnosis and procedure codes for identifying colon and lung cancer surgical Medicare FFS beneficiaries

Type of code	Codes
**Colon**	
ICD-9 diagnosis codes	1530, 1531, 1532, 1533, 1534, 1536, 1537, 1538, 1539, 1540
ICD-10 diagnosis codes	C18.0, C18.2, C18.3, C18.4, C18.5, C18.6, C18.7, C18.8, C18.9, C19.0
Cancer-directed surgery: CPT codes	44140, 44141, 44143, 44144, 44145, 44146, 44147, 44150, 44151, 44152, 44153, 44155, 44156, 44157, 44158, 44160, 44204, 44205, 44206, 44207, 44208, 44210, 44211, 44212, 45112, 45113, 45114, 45116, 45119, 45120, 45121, 45123, 45126
Cancer-directed surgery: ICD-9-PCS	4571, 4572, 4573, 4574, 4575, 4576, 4579, 4581, 4582, 4583
Cancer-directed surgery: ICD-10-PCS	0DTE4ZZ, 0DTF4ZZ, 0DTG4ZZ, 0DTH4ZZ, 0DTK4ZZ, 0DTL4ZZ, 0DTM4ZZ, 0DTN4ZZ, 0DTE8ZZ, 0DTF8ZZ, 0DTG8ZZ, 0DTH8ZZ, 0DTK8ZZ, 0DTL8ZZ, 0DTM8ZZ, 0DTN8ZZ, 0DTEFZZ, 0DTFFZZ, 0DTGFZZ, 0DTHFZZ, 0DTKFZZ, 0DTLFZZ, 0DTMFZZ, 0DTNFZZ, 0DTE0ZZ, 0DTF0ZZ, 0DTG0ZZ, 0DTH0ZZ, 0DTK0ZZ, 0DTL0ZZ, 0DTM0ZZ, 0DTN0ZZ, 0DTE7ZZ, 0DTF7ZZ, 0DTG7ZZ, 0DTH7ZZ, 0DTK7ZZ, 0DTL7ZZ, 0DTM7ZZ, 0DTN7ZZ, 8E0W0CZ, 8E0W3CZ, 8E0W4CZ, 8E0W7CZ, 8E0W8CZ
**Lung**	
ICD-9 diagnosis codes	1622, 1623, 1624, 1625, 1628, 1629
ICD-10 diagnosis codes	C34.2, C39.9, C34.00, C34.01, C34.02, C34.10, C34.11, C34.12, C34.20, C34.30, C34.31, C34.32, C34.80, C34.81, C34.82, C34.90, C34.91, C34.92
Cancer-directed surgery: CPT codes	32440, 32442, 32445, 43480, 32482, 32484, 32486, 32488, 32503, 32504, 32505, 32506, 32507, 32520, 32522, 32657, 32663, 32666, 32667, 32668, 32669, 32670, 32671
Cancer-directed surgery: ICD-9-PCS	321, 326, 329, 3209, 3220, 3229, 3230, 3239, 3241, 3249, 3250, 3259
Cancer-directed surgery: ICD-10-PCS	0BB30ZZ, 0BB33ZZ, 0BB40ZZ, 0BB43ZZ, 0BB50ZZ, 0BB53ZZ, 0BB60ZZ, 0BB63ZZ, 0BB70ZZ, 0BB73ZZ, 0BB80ZZ, 0BB83ZZ, 0BB90ZZ, 0BB93ZZ, 0BBB0ZZ, 0BBB3ZZ, 0BBC0ZZ, 0BBC3ZZ, 0BBD0ZZ, 0BBD3ZZ, 0BBF0ZZ, 0BBF3ZZ, 0BBG0ZZ, 0BBG3ZZ, 0BBH0ZZ, 0BBH3ZZ, 0BBJ0ZZ, 0BBJ3ZZ, 0BBK0ZZ, 0BBK3ZZ, 0BBL0ZZ, 0BBL3ZZ, 0BBM0ZZ, 0BBM3ZZ

ICD = International Classification of Diseases; CPT = Current Procedural Terminology; PCS = Procedure Coding System.

© 2026 Kim M et al. *JAMA Network Open*.

eTable 2. List of complications following cancer-directed surgery

**Conditions included in the 90-day post-operative complications measure**
Post-operative complications related to urinary tract anatomy (renal failure)
Post-operative pulmonary compromise
Post-operative acute myocardial infarction
Post-operative pneumonia
Post-operative venous thrombosis and pulmonary embolism
Post-operative hemorrhage or hematoma
Post-operative wound infection
Post-operative dementia/delirium
Post-operative gastrointestinal hemorrhage or ulceration following non-gastrointestinal surgery

© 2026 Kim M et al. *JAMA Network Open*.

eTable 3. Colon—Sensitivity analysis for 90-day mortality

	(1) Full sample (main analysis)<br><br>Odds ratio (95% CI)	(2) Surgery within 2 years of closure<br><br>Odds ratio (95% CI)	(4) Surgery within 4 years of closure<br><br>Odds ratio (95% CI)	(6) Surgery within 6 years of closure<br><br>Odds ratio (95% CI)
Unadjusted model	**1.32***<br>(1.22, 1.42)**	1.09<br>(0.94, 1.28)	**1.19**<br>(1.06, 1.33)**	**1.24***<br>(1.12, 1.36)**
Adjusted model	**1.11*<br>(1.01, 1.22)**	0.99<br>(0.83, 1.18)	1.04<br>(0.91, 1.19)	1.06<br>(0.95, 1.18)
N beneficiaries treated at hospitals that did not close	354,546	354,546	354,546	354,546
N beneficiaries treated at closing hospitals	6,018	1,684	2,971	4,099
MDES with given sample sizes, alpha = 0.05, power = 0.80	1.13	1.24	1.18	1.15

CI = confidence interval; MDES = minimum detectable effect size; N = number
* p<0.05, ** p<0.01, *** p<0.001.

eTable 4. Colon—Sensitivity analysis for 90-day complications

	(1) Full sample (main analysis)<br><br>Odds ratio (95% CI)	(2) Surgery within 2 years of closure<br><br>Odds ratio (95% CI)	(4) Surgery within 4 years of closure<br><br>Odds ratio (95% CI)	(6) Surgery within 6 years of closure<br><br>Odds ratio (95% CI)
Unadjusted model	**1.30***<br>(1.23, 1.37)**	**1.13*<br>(1.03, 1.25)**	**1.25***<br>(1.16, 1.34)**	**1.26***<br>(1.19, 1.34)**
Adjusted model	**1.10*<br>(1.01, 1.21)**	1.02<br>(0.90, 1.16)	1.09<br>(0.98, 1.22)	1.08<br>(0.98, 1.20)
N beneficiaries treated at hospitals that did not close	354,546	354,546	354,546	354,546
N beneficiaries treated at closing hospitals	6,018	1,684	2,971	4,099
MDES with given sample sizes, alpha = 0.05, power = 0.80	1.08	1.15	1.11	1.09

CI = confidence interval; MDES = minimum detectable effect size; N = number
* p<0.05, ** p<0.01, *** p<0.001.

© 2026 Kim M et al. *JAMA Network Open*.

eTable 5. Colon—Sensitivity analysis for length of stay

	(1)<br>Full sample<br>(main analysis)<br><br>OLS coef.<br>(95% CI)	(2)<br>Surgery within 2<br>years of closure<br><br>OLS coef.<br>(95% CI)	(4)<br>Surgery within 4<br>years of closure<br><br>OLS coef.<br>(95% CI)	(6)<br>Surgery within 6<br>years of closure<br><br>OLS coef.<br>(95% CI)
Unadjusted model	**1.18*****<br>**(1.00, 1.37)**	**0.66*****<br>**(0.32, 1.00)**	**0.79*****<br>**(0.53, 1.05)**	**0.92*****<br>**(0.70, 1.15)**
Adjusted model	0.11<br>(-0.27, 0.50)	0.07<br>(-0.44, 0.58)	-0.00<br>(-0.42, 0.42)	0.03<br>(-0.35, 0.41)
N beneficiaries treated at hospitals that did not close	354,546	354,546	354,546	354,546
N beneficiaries treated at closing hospitals	6,018	1,684	2,971	4,099
MDES with given sample sizes, alpha = 0.05, power = 0.80	0.24	0.44	0.33	0.28

Coef. = coefficient; CI = confidence interval; MDES = minimum detectable effect size; N = number; OLS = ordinary least square
* p<0.05, ** p<0.01, *** p<0.001.

eTable 6. Lung—Sensitivity analysis for 90-day mortality

	(1)<br>Full sample<br>(main analysis)<br><br>Odds ratio<br>(95% CI)	(2)<br>Surgery within 2<br>years of closure<br><br>Odds ratio<br>(95% CI)	(4)<br>Surgery within 4<br>years of closure<br><br>Odds ratio<br>(95% CI)	(6)<br>Surgery within 6<br>years of closure<br><br>Odds ratio<br>(95% CI)
Unadjusted model	**1.58*****<br>**(1.35, 1.85)**	0.87<br>(0.60, 1.27)	1.08<br>(0.83, 1.41)	**1.35****<br>**(1.11, 1.66)**
Adjusted model	1.26<br>(0.96, 1.64)	0.76<br>(0.45, 1.27)	0.91<br>(0.63, 1.32)	1.14<br>(0.80, 1.61)
N beneficiaries treated at hospitals that did not close	196,206	196,206	196,206	196,206
N beneficiaries treated at closing hospitals	1,938	557	972	1,318
MDES with given sample sizes, alpha = 0.05, power = 0.80	1.29	1.56	1.42	1.35

CI = confidence interval; MDES = minimum detectable effect size; N = number
* p<0.05, ** p<0.01, *** p<0.001.

© 2026 Kim M et al. *JAMA Network Open*.

eTable 7. Lung—Sensitivity analysis for 90-day complications

	(1)<br>Full sample<br>(main analysis)<br><br>Odds ratio<br>(95% CI)	(2)<br>Surgery within 2<br>years of closure<br><br>Odds ratio<br>(95% CI)	(4)<br>Surgery within 4<br>years of closure<br><br>Odds ratio<br>(95% CI)	(6)<br>Surgery within 6<br>years of closure<br><br>Odds ratio<br>(95% CI)
Unadjusted model	**1.68***<br>(1.53, 1.83)**	**1.43***<br>(1.21, 1.69)**	**1.42***<br>(1.25, 1.61)**	**1.55***<br>(1.39, 1.72)**
Adjusted model	**1.43***<br>(1.17, 1.76)**	**1.36*<br>(1.02, 1.81)**	1.28<br>(0.97, 1.70)	**1.38*<br>(1.08, 1.77)**
N beneficiaries treated at hospitals that did not close	196,206	196,206	196,206	196,206
N beneficiaries treated at closing hospitals	1,938	557	972	1,318
MDES with given sample sizes, alpha = 0.05, power = 0.80	1.14	1.27	1.20	1.17

CI = confidence interval; MDES = minimum detectable effect size; N = number
* p<0.05, ** p<0.01, *** p<0.001.

eTable 8. Lung—Sensitivity analysis for length of stay

	(1)<br>Full sample<br>(main analysis)<br><br>OLS coef.<br>(95% CI)	(2)<br>Surgery within 2<br>years of closure<br><br>OLS coef.<br>(95% CI)	(4)<br>Surgery within 4<br>years of closure<br><br>OLS coef.<br>(95% CI)	(6)<br>Surgery within 6<br>years of closure<br><br>OLS coef.<br>(95% CI)
Unadjusted model	**1.36***<br>(1.08, 1.64)**	0.48<br>(-0.44, 1.00)	**0.52**<br>(0.12, 0.91)**	**0.72***<br>(0.38, 1.06)**
Adjusted model	0.65<br>(-0.25, 1.56)	0.09<br>(-0.93, 1.10)	0.06<br>(-0.98, 1.10)	0.25<br>(-0.70, 1.19)
N beneficiaries treated at hospitals that did not close	196,206	196,206	196,206	196,206
N beneficiaries treated at closing hospitals	1,938	557	972	1,318
MDES with given sample sizes, alpha = 0.05, power = 0.80	0.39	0.72	0.55	0.47

Coef. = coefficient; CI = confidence interval; MDES = minimum detectable effect size; N = number; OLS = ordinary least square
* p<0.05, ** p<0.01, *** p<0.001.

© 2026 Kim M et al. *JAMA Network Open*.

eFigure. Sample flowchart

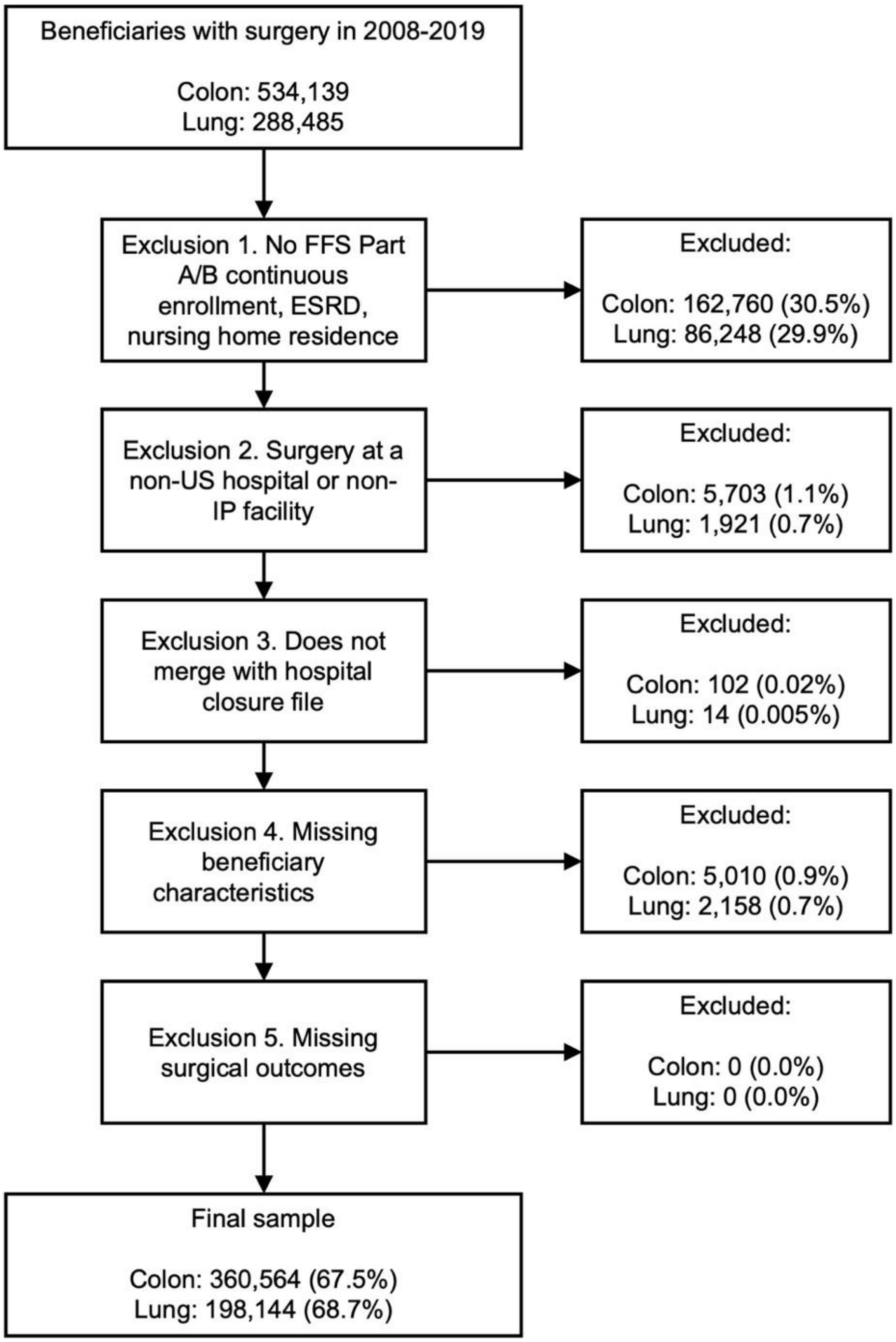

© 2026 Kim M et al. *JAMA Network Open*.
