# Supplementary material for: Outcomes Among Medicare Beneficiaries After Cancer Surgery in Hospitals That Subsequently Closed
Source: JAMA Netw Open. 2026 Jan 13;9(1):e2553704. doi: 10.1001/jamanetworkopen.2025.53704 (PMC12801090; doi:10.1001/jamanetworkopen.2025.53704)
Supplement: Supplement 2. — Data Sharing Statement [file jamanetwopen-e2553704-s002.pdf]

Data Sharing Statement

Kim. Outcomes Among Medicare Beneficiaries After Cancer Surgery in Hospitals That Subsequently Closed. *JAMA Netw Open*. Published January 13, 2026. doi:10.1001/jamanetworkopen.2025.53704

Data
Data available: No

Additional Information
Explanation for why data not available: CMS data use agreement restricts data sharing.
